# DNA Methylation and Potential for Epigenetic Regulation in *Pygospio elegans*

**DOI:** 10.1371/journal.pone.0151863

**Published:** 2016-03-23

**Authors:** Jenni E. Kesäniemi, Liisa Heikkinen, K. Emily Knott

**Affiliations:** Department of Biological and Environmental Science, University of Jyväskylä, Jyväskylä, Finland; Inc, UNITED STATES

## Abstract

Transitions in developmental mode are common evolutionarily, but how and why they occur is not understood. Developmental mode describes larval phenotypes, including morphology, ecology and behavior of larvae, which typically are generalized across different species. The polychaete worm *Pygospio elegans* is one of few species polymorphic in developmental mode, with multiple larval phenotypes, providing a possibility to examine the potential mechanisms allowing transitions in developmental mode. We investigated the presence of DNA methylation in *P*. *elegans*, and, since maternal provisioning is a key factor determining eventual larval phenotype, we compared patterns of DNA methylation in females during oogenesis in this species. We demonstrate that intragenic CpG site DNA methylation and many relevant genes necessary for DNA methylation occur in *P*. *elegans*. Methylation-sensitive AFLP analysis showed that gravid females with offspring differing in larval developmental mode have significantly different methylation profiles and that the females with benthic larvae and non-reproductive females from the same location also differ in their epigenetic profiles. Analysis of CpG sites in transcriptome data supported our findings of DNA methylation in this species and showed that CpG observed/expected ratios differ among females gravid with embryos destined to different developmental modes. The differences in CpG site DNA methylation patterns seen among the samples suggest a potential for epigenetic regulation of gene expression (through DNA methylation) in this species.

## Introduction

Epigenetic changes can affect the regulation of gene expression and increase variation at the phenotypic level without changing the underlying DNA sequence. The most commonly studied mechanism of epigenetic change is DNA methylation, which involves addition of a methyl group to a cytosine nucleotide (predominantly at CpG dinucleotide sites), altering its structure to 5-methylcytosine (5mC). DNA methylation has significant roles in many biological processes, including cell type differentiation during embryonic development, X-chromosome inactivation, regulation of genome stability and silencing of transposable elements (reviewed by [1–4]). DNA methylation was first linked to regulation of gene expression in studies on mammals, when methylation in promoter regions of genes was shown to silence the expression of those genes. More recently, mainly intragenic methylation has been observed in invertebrates, which is suggested to affect gene expression and for example, alternative splicing [3, 5–8].

DNA methylation (and likewise demethylation, the removal of methyl groups from CpG sites) as a mechanism for regulating gene expression has been suggested to be a particularly important means for organisms to adapt to new or varying environments or to maintain phenotypic plasticity in some traits (reviewed by [9]). This is because some of the DNA methylation patterns are expected to be transient and more easily modified by environmental factors than are changes to DNA itself. Multiple examples of methylation patterns being affected by environmental factors can be found. Temperature stress caused changes in global methylation patterns in an Antarctic polychaete [10], and short term changes in global methylation patterns were seen in hatchery reared brown trout when feeding them a salt-enriched diet [11]. Moreover, in *Arabidopsis thaliana*, Dubin and colleagues [12] found a correlation between methylation levels and latitude (and other environmental variables) and pathogen related stress has been shown to induce changes in DNA methylation and gene expression patterns [13].

Several experiments with plants have shown a connection between methylation or demethylation patterns and phenotypic differences (e.g. stem elongation plasticity and shade avoidance response [14] and flowering time [15]). Studies on variation in methylation patterns in different ecotypes or phenotypes of animal populations are less common. However, intragenic methylation patterns were linked with gene expression regulation and resistance to insecticides in the peach-potato aphid [5] and in Sea bass, sex ratio is affected by temperature through changes in methylation of promoter regions [16]. Additionally, the marine and freshwater morphotypes of threespine stickleback, *Gasterosteus aculeatus*, have differences in their genome-wide methylation patterns, and epigenetic regulation (through DNA methylation) is suggested to be associated with the phenotypic differences seen between the morphs [17].

Our study species, the spionid polychaete *Pygospio elegans*, is polymorphic in an important life history characteristic: larval developmental mode (such variation is termed poecilogony). Marine invertebrate species normally produce only one type of larva, but *P*. *elegans* is one of the rare poecilogonous species, having benthic, planktonic and intermediate larval modes. Females brood their larvae in egg capsules within the sand tubes they inhabit, and differential maternal provisioning is associated with different larval developmental modes. The types of larvae differ in e.g. brood size, larval size, and larval dispersal ability, i.e. length of pelagic period. Benthic larvae develop when mothers lay only a few embryos, but abundant nutritional resources (yolky nurse eggs) in their egg capsules. These larvae have a long brooding period and settle soon after they emerge from the egg capsules. Planktonic larvae, on the other hand, emerge from the egg capsules early in development to feed and grow in the plankton before settlement as juveniles. Mothers of planktonic larvae lay many embryos and fewer nurse eggs in the capsules. Intermediates between these two extremes also exist [18, 19].

The underlying cause of the observed polymorphism in developmental mode in *P*. *elegans* is not known, but epigenetic regulation (e.g. through DNA methylation) could be one contributing factor. Although some genetic structuring is present among populations, it does not appear to correlate with developmental mode and there is no evidence for cryptic species or lineages with different developmental modes [19, 20, 21]. Mothers producing different types of larvae could differ in their DNA methylation patterns and therefore also gene expression patterns, allowing polymorphism in development without genetic differentiation. It is not known if individual females can produce multiple types of larvae, but in some populations, multiple types of larvae have been observed either simultaneously or seasonally ([21, 22], unpublished observations).

To begin to address the question of whether epigenetic processes could be involved in the developmental polymorphism observed in *P*. *elegans*, we studied DNA methylation in this species. First, we confirmed that DNA methylation at CpG sites occurs in *P*. *elegans* using bisulfite treatment and DNA sequencing of a single locus. Secondly, genome-wide methylation patterns were compared in gravid females producing different types of larvae using restriction enzymes with different sensitivity to methylation. Additionally, we searched for genes suggested to be involved in the methylation machinery from the transcriptome of *P*. *elegans*. Finally, we performed an analysis of the observed/expected ratio of CpG sites in the transcriptome in order to investigate the evolutionary signature of methylation in *P*. *elegans* and to compare levels of intragenic germline DNA methylation in different functional groups of genes.

## Methods

### Confirmation of CpG site methylation

To investigate the presence of CpG site DNA methylation in *P*. *elegans*, we used bisulfite treatment and DNA sequencing of a specific gene fragment. This method can be used to detect methylation at single base resolution. Bisulfite treatment of DNA converts unmethylated cytosines to uracil while methylated cytosines remain unchanged. After sequencing, the converted sites can be detected as thymines in the sequences. *P*. *elegans* samples were collected in March 2014 from Vellerup, Denmark (southern Baltic Sea). Two adult females and five samples of larvae at different developmental stages (early stage embryos before organogenesis, and late brooded larvae with multiple segments) were used. DNA was extracted using the DNeasy blood and tissue kit spin column protocol (Qiagen). Portions of the same DNA samples were bisulfite treated using the EZ DNA Methylation kit (Zymo Research) according to the manufacturer’s instructions. A fragment of the Histone H3 gene was amplified and sequenced from both untreated and bisulfite treated DNA samples. For amplification of untreated DNA, we used primers published previously (H3F and H3R, [23]). Due to the changes in the sequence after the bisulfite treatment, new primers were designed using the Bisulfite Primer Seeker 12S (Zymo Research) for use in amplification and sequencing of the bisulfite treated DNA: H3bs F: 5’- TTAGTTATYGGGGGAGTTAAGAAGTTTTATAGG -3’, H3bs R: 5’-ACACRCTTAACATAAATAACACACAAATTAATATC -3’. PCR was conducted in 20 μl reactions with 1X PCR Buffer (Biotools), 1.5 mM MgCl_2_, 200 μM of dNTPs, 0.5 μM of each primer, and 0.1 U of Taq polymerase (Biotools). For amplification, the following PCR program was used: 94°C for 2 min, 34 cycles of 94°C for 30 sec, 60°C for 30 sec and 72°C for 30 sec, with a final elongation of 72°C for 3 min. PCR products were treated with Exonuclease I and Shrimp alkaline phosphatase (Fermentas), cycle sequenced in both directions using the BigDye v.3.1 kit, and visualized with an ABI 3130xl Genetic Analyzer and Sequencing Analysis v.6 software (Applied Biosystems). Sequences were checked and alignments of sequences obtained from both untreated and bisulfite treated DNA were made using Sequencer v.5.2.4 (Gene Codes Corporation) to investigate the methylation status of the cytosines.

### Genome-wide methylation patterns

After confirming the presence of CpG methylation in *P*. *elegans*, methylation patterns and epigenetic variation was examined using the methylation-sensitive AFLP (MS-AFLP) technique [24], modified from the standard AFLP protocol [25]. This method is based on comparing patterns (restriction fragments) produced using two restriction enzymes, *Msp*I and *Hpa*II, which have the same recognition site (5’- CCGG -3’) but different sensitivity to methylation. *Msp*I is insensitive to methylation of the CpG site in the recognition sequence, but *Hpa*II activity is inhibited if the inner cytosine is methylated. With this method, no genome sequence information is needed, and the resulting banding patterns indicate differentially methylated loci in the genome.

We used the MS-AFLP method to estimate levels of methylation and epigenetic differentiation of females producing different types of larvae. We sampled gravid and non-reproductive females in May 2014 from populations that produce either benthic larvae or planktonic larvae: Herslev, Denmark (Southern Baltic Sea), and Rømø, Denmark (Wadden Sea), respectively. Each genomic DNA sample was digested in parallel reactions with *Eco*RI and either *Msp*I or *Hpa*II enzyme, which, as described earlier, are isoschizomers that differ in their sensitivity to CpG site methylation.

Individual worms were first macerated in 180 μl of ATL Buffer and 20 μl of Proteinase K (Qiagen) and then incubated at 56°C for three hours, after which DNA extraction was completed using the Genomic DNA Clean and Concentrator kit (Zymo Research) according to the manufacturer’s instructions. For MS-AFLP, 300 ng of genomic DNA was used per reaction. Parallel digestions were done in 20 μl reactions containing 1 μl of *Eco*RI and 1 μl of either *Msp*I or *Hpa*II (FastDigest enzymes, all Thermo Scientific). Samples were digested at 37°C for 15 min, followed by 5 min both at 65°C and then 80°C.

Double stranded ligation adaptors were prepared separately by mixing equal amounts of the oligonucleotides (*Eco*RI: 5’- AATTGGTACGCAGTCTAC -3’ and 5’- CTCGTAGACTGCGTACC -3’ [25]; *Hpa*II/*Msp*I: 5’- GACGATGAGTCTAGAA -3’ and 5’- CGTTCTAGACTCATC -3’ [26]) and heating them at 95°C for 5 min, after which they were cooled to ambient temperature. The adapters were then ligated to the digested DNA from our samples. A mixture containing 100 pmol of the *Eco*RI adaptor, 15 pmol of the *Hpa*II/*Msp*I adaptor, and 2 U of T4 DNA ligase (Thermo Scientific) was first prepared, and 5 μl was added to each 20 μl digestion sample (described above) and incubated at 18°C overnight (16 hours). Afterwards, the ligase enzyme was inactivated at 65°C for 10 min.

Preselective PCR contained 2 μl of the prepared ligation mix as template, 0.5 μM of both preselective primers (Pre-EcoRI: 5’- GACTGCGTACCAATTCA -3’, Pre-HpaII/MspI: 5’- GATGAGTCTAGAACGGT -3’ [26]), 200 μM of dNTPs, 0.2 U of Q5 High-Fidelity DNA polymerase (New England BioLabs) and 1X of Q5 Reaction Buffer (with 2 mM MgCl_2_) in 10 μl volume. The following PCR program was used: 72°C for 2 min, 30 cycles of 95°C for 30 s, 59°C for 30 s, and 72°C for 2 min, with a final extension at 60°C for 30 min. Six different primer combinations (Table C in S1 File) were used in selective PCRs. PCR was conducted in 10 μl volume using 1 μl of (1:10 diluted) preselective PCR product as template, 0.5 μM of selective primers (EcoRI fluorescently labeled with FAM), 200 μM of dNTPs, 0.25 U of DreamTaq DNA polymerase (Fermentas) and 1X of DreamTaq Buffer (with final MgCl_2_ concentration of 2 mM). PCR cycling conditions were as follows: 72°C for 2 min, 13 cycles of 95°C for 30 s, 65°C for 30 s (decreasing temperature 0.7°C/cycle), and 72°C for 2 min, followed by 23 cycles of 95°C for 30 s, 57°C for 30 s and 72°C for 1 min (with an extension of time by 2 sec/cycle), ending with 72°C for 20 min. The resulting PCR products were separated on an ABI PRISM 3130xl (using GeneScan -500 LIZ as size standard), and fragments between 50 and 500 bp were scored using GeneMapper v.5 software (Applied Biosystems).

Presence (1) and absence (0) of fragments was scored for each individual in the two parallel reactions (*Eco*RI-*Msp*I and *Eco*RI-*Hpa*II), with four possible resulting patterns: 1/1, 1/0, 0/1 and 0/0. In an unmethylated state, the fragment is seen in both reactions (pattern 1/1), whereas absence of both fragments (no digestion, 0/0) is considered an uninformative state, which could be due to either hypermethylation or absence of the restriction site [27, 28, 29]. In a methylated state, fragments are seen in only one of the reactions. The full methylation state, when the inner cytosine of the recognition site is methylated on one or both strands, is coded as 1/0 (*Msp*I band present, *Hpa*II blocked by methylation, band absent). The interpretation of the 0/1 pattern (*Msp*I band absent, *Hpa*II band present) remains ambiguous, and in some cases [e.g. 11, 27, 28, 29], this is considered to be a hemimethylated state, in which the external cytosine of the recognition site is methylated on one of the strands. However, we decided to not to consider this pattern as a case of hemimethylation, since based on information from the enzyme manufacturer (Thermo Scientific), both enzymes are unable to cleave the recognition site when the external cytosine is methylated. Additionally, methylation of external cytosines is not in the context of CpG site methylation, therefore, we will refer to pattern 0/1 as unspecific products. Methylation profiles (presence/absence fragment patterns) were compared to investigate differences among groups: between gravid and non-reproductive females within sites, and between sites. Before further analyses, the data was transformed to combine the two lines of data/individual (*Eco*RI-*Msp*I and *Eco*RI-*Hpa*II profiles) into one line of binary data/individual, and two different scoring approaches were used (described below).

Data was first transformed and analyzed using the R package msap [27]. Here, every locus (fragment) was classified as either methylation-susceptible (MSL) or nonmethylated (NML), depending on the observed proportion of methylated states across samples exceeding the 5% default error rate-based threshold [27], and only the MSL loci were used to examine epigenetic variation in msap (MSL loci include both the 1/0 and the 0/1 patterns, described above). Frequencies of genome-wide methylation and absence of methylation were estimated for the four groups of samples. Epigenetic (MSL) differentiation was estimated using AMOVA (10 000 permutations). In addition, pairwise epigenetic differentiation (MSL) was estimated using AMOVA based Φ_ST_ analyses.

Epigenetic diversity was also examined by transforming the data with another scoring method using the R script MSAP_calc [28]. Since the msap program defines methylation-susceptible loci (MSL) by combining both methylated and the proposed hemimethylated loci together, we also used the ‘Mixed Scoring 2’ strategy [28] to transform the data into three different marker types: condition I indicates the unmethylated state (u: fragment present in both reactions, *Msp*I & *Hpa*II), condition II indicates methylation of the internal cytosine(s) (m: band present in *Msp*I reaction only) and condition III represents either hemimethylation of the external cytosine or unspecific products (h: band present in *Hpa*II reaction only). These three conditions are respectively, the 1/1, 1/0 and 0/1 patterns described above. Absence of fragments in both reactions indicates an uninformative state. During data transformation, one to three subloci (or epiloci) for each locus (restriction site) of the raw data are generated, i.e. one original locus can contain three epiloci (u, m, h). This data was used to measure epigenetic diversity within the samples by calculating number and percentage of polymorphic epiloci of each type in the samples, percentage of private epiloci (pattern seen in just one sample) and mean Shannon’s information index (see [28]). Differentiation of the methylation profiles among samples was also estimated with AMOVA (pairwise PhiPT, an F_ST_ analogue for codominant and binary data, AMOVA) in GenAlEx [30], using the methylated epiloci only (m, see above).

### CpG observed/expected ratio analysis

DNA methylation at CpG sites leads to gradual depletion of CpG dinucleotides in genomes over evolutionary time [31, 32]. This is because methylated cytosines are vulnerable to deamination (spontaneous mutation to thymines), resulting in a lower than expected amount of CpG dinucleotides in sequences that are highly and consistently methylated. The relationship between the observed and expected frequency of CpG dinucleotides, the CpG o/e ratio, can be calculated from DNA sequence data and allows for an estimation of levels of DNA methylation in a genome or expressed genes. Low CpG o/e ratio is an indication of high germline methylation, whereas high CpG o/e ratio indicates DNA sequences with less methylation. For CpG o/e ratio analysis, draft transcriptome data from *P*. *elegans* (Heikkinen et al. in prep.) was utilized. The data are pooled from multiple Illumina RNAseq paired-end libraries (total of ~300M read pairs) obtained from: gravid females that produce planktonic larvae (2 individuals), gravid females that produce benthic larvae (2 individuals) and non-reproductive females from populations that produce both planktonic and benthic larvae (2 individuals). The transcriptome is expected to be suitable for evaluation of the methylation status of *P*. *elegans’* DNA because earlier studies with other invertebrate species have shown that methylation is typically seen in coding regions [7, 33], and predominantly in exons [34, 35, 36]. However, in addition to protein coding sequences, the draft transcriptome assembly contains also long non-coding RNAs. To excude the non-coding RNA from the analysis, we restricted our study to contigs which included predicted open reading frames (ORFs).

In total, the draft transcriptome of *P*. *elegans* contains 80853 contigs presenting predicted open reading frames (ORFs). GO terms were linked to ORFs according to their best BLAST hit in Swiss-Prot or TrEMBL database (e-value treshold 1.0E-06) and PfamA domain hits. In order get an as reliable as possible set of transcripts for evaluation of the difference in CpG o/e ratio among gene functional classes, we chose 8658 ORF containing contigs annotated with a biological process GO term and expressed with a minimum average of 10 fragments per kilobase of exon per million fragments mapped (FPKM) in at least one of the studied conditions (gravid females that produce planktonic larvae, gravid females that produce benthic larvae, non-reproductive females). The CpG observed/expected ratio (CpG o/e) was calculated for each contig in this set using the following equation (*l* is contig length; the number of nucleotides):

$$\mathrm{CpGo}/e=\frac{\mathrm{number of CpG}}{\mathrm{number of C}\times \mathrm{number of G}}\times \frac{l^{2}}{l-1}$$

Additionally, each of the ORF containing contigs were assigned to a functional group based on the mgi GO Slim Categories biological process (mgi is the international database resource for the laboratory mouse: http://www.informatics.jax.org). Because multiple GO terms are typically associated to each contig, the same contig might have been assigned to many GO Slim bins. However, any single contig was allowed to be assigned only once to a specific GO Slim bin. For each GO Slim category, we calculated the mean CpG o/e and standard error of the mean. The differences among the category means were analyzed with one way ANOVA followed by pairwise comparison using t-tests with pooled standard deviation. Division to GO Slim categories was applied separately for the subgroups of contigs expressed in the three study groups. The differences between mean CpG o/e in different GO Slim categories were analyzed with t-tests (comparing gravid females with benthic larvae vs. gravid females with planktonic larvae). All statistical testing was conducted in R (R Core Team, www.R-project.org/).

### Genes involved in the DNA methylation machinery

We searched for genes expected to be involved in the molecular machinery of DNA methylation from a draft transcriptome of *P*. *elegans* (unpublished). Transcriptome data were obtained from multiple individuals (gravid females of both developmental types, as well as non-reproductive individuals, see above). We attempted to find homologs of genes from the relevant protein families: DNA methyltranseferases (Dnmt, focusing on *Dnmt1* and 3 since *Dnmt2* does not have DNA methylation function) and Methyl-CpG Binding Domain proteins (MBD). DNA methyltransferases are a well characterized family of proteins involved in establishing and maintaining DNA methylation of cytosines (see [6, 37]). Methyl-CpG binding proteins (MBDs) bind to methylated CpG sites and are involved in repression of gene expression [6, 9, 38, 39]. Additionally, we also searched for possible presence of Ten Eleven Translocation enzymes (TET). In addition to having other functions in mammals, these enzymes are suggested to be involved in demethylation processes (mammalian studies: [40, 41]), by catalyzing the conversion of methylated cytosine (5mC) to 5-hydroxymethylcytosine (5hmC). Blastp searches were done against the draft *P*. *elegans* transcriptome contig database, using sequences from *Homo sapiens* as query (see S2 File). Once matches to genes of interest were found, the *P*. *elegans* contigs were then used to search for conserved regions and identify protein domains using NCBI’s Conserved Domain search tool (http://www.ncbi.nlm.nih.gov/Structure/cdd/wrpsb.cgi). Protein translations of the matched *P*. *elegans* contigs were also blasted (blastp, nr & refseq_protein databases) to confirm gene identity (http://blast.ncbi.nlm.nih.gov/Blast.cgi). In addition, phylogenetic reconstructions were used to give further support for the orthology of the gene sequences. Sequence alignments with known representative sequences and best blast hits of the contigs were done in MEGA6 [42], and Neighbor-Joining (NJ) phylogenetic trees were constructed using MEGA6 (1 000 bootstrap replicates).

## Results

### Bisulfite sequencing

Bisulfite treatment and sequencing of a fragment of the Histone H3 gene was used to reveal the presence of CpG site DNA methylation in *P*. *elegans*. A 367 bp fragment of the gene was sequenced from genomic DNA and the sequences of all seven samples were identical (as well as identical to H3 sequence for *P*. *elegans* available in GenBank KJ747057.1). After bisulfite conversion and sequencing with newly designed primers, the resulting fragments (191–230 bp), were aligned with the data from the untreated DNA (Fig A in S1 File). Majority of cytosines were converted to thymines during bisulfite treatment, however, eight cytosines (12%, all at CpG sites) were not converted, indicating methylation at those sites. The same CpG sites were methylated in all seven samples, which confirms that methylation within a coding region is possible in this species.

### Methylation-sensitive AFLP

Methylation-sensitive AFLP (MS-AFLP) protocol was used for analyses of genome wide methylation levels and to compare methylation profiles between females producing embryos destined to different larval developmental modes. In the MS-AFLP protocol, each DNA sample is digested in separate parallel reactions using *Hpa*II and *Msp*I, restriction enzymes with the same recognition sequence (containing a CpG site), but different sensitivity to methylation. Methylation profiles for each individual can be constructed by comparing the fragments produced by the different enzymes.

Six primer combinations produced altogether 165 fragments (loci from now on). Using msap [27], and methylation scoring procedures outlined by [27, 29], 105 loci were classified as methylation-sensitive loci (MSL), and 101 of these (96%) were polymorphic. Generally, the frequencies of methylation (CpG site methylated) were higher in gravid females (Herslev: 0.265; Rømø: 0.269) compared to non-reproductive females in both sites (Herslev: 0.212; Rømø: 0.243) (Table 1). Some differences in the genome-wide methylation patterns were seen among all groups (MSL loci AMOVA, Φ_ST_ = 0.057, P < 0.001). Most pairwise comparisons of the methylation profiles showed significant differences (Table 2). Within the Herslev population, non-reproductive and gravid females showed significant differences in their methylation profiles (MSL Φ_ST_ = 0.064, P = 0.013), whereas in Rømø, gravid females did not differ from non-reproductive females (MSL Φ_ST_ = -0.034, P = 0.898). Gravid females producing different larval types (Herslev: benthic & Rømø: planktonic) also showed significant differences in their MSL loci (MSL Φ_ST_ = 0.057, P = 0.021). In addition, also the non-reproductive females from the two sites were significantly differentiated in their methylation profiles (Table 2).

**Table 1:** Frequencies (%) of the different states of methylation in the samples.

Methylation state (band pattern, *Msp*I/*Hpa*II)	Herslev_B (N = 18)	Herslev_nr (N = 12)	Rømø_P (N = 12)	Rømø_nr (N = 12)
Unmethylated (1/1)	0.181	0.178	0.241	0.253
Methylated (internal cytosine) (1/0)	0.265	0.212	0.269	0.243
Unspecific (hemimethylated) (0/1)	0.025	0.022	0.055	0.053
Uninformative* (0/0)	0.529	0.588	0.435	0.451

Methylation sensitive MS-AFLP loci (MSL) were used [27]

B = benthic, P = planktonic, nr = non-reproductive, N = number of individuals in the sample

*absence of target or hypermethylation

**Table 2:** Pairwise AMOVA estimations (Φ_ST_/PhiPT) between all groups.

	Herslev_B	Herslev_nr	Rømø_P	Rømø_nr
**Herslev_B**		0.013/0.002	0.021/<0.001	<0.001/<0.001
**Herslev_nr**	**0.064**/**0.051**		0.161/0.003	0.004/<0.001
**Rømø_P**	**0.057/0.064**	0.030/**0.060**		0.898/0.455
**Rømø_nr**	**0.105/0.065**	**0.099/0.077**	-0.034/-0.008	

Φ_ST_ for methylation-sensitive loci (MSL)/PhiPT for Mixed scoring 2 markers (methylated epiloci, m) below diagonal (bolded values with P < 0.05), p-values above diagonal (B = benthic, P = planktonic, nr = non-reproductive)

An alternative scoring method, the mixed scoring transformation (‘Mixed Scoring 2’, [28]) of the data revealed altogether 395 markers or epiloci. Analyses with this scoring of the data produced similar results as the first scoring method, described above. AMOVA suggested epigenetic differences among groups (PhiPT = 0.049, P <0.001) and all pairwise comparisons were significant except between gravid and non-gravid females from Rømø, which did not show differences in their methylation profiles (Table 2). Epigenetic diversity was highest in Rømø (in % polymorphic markers & private markers and Shannon’s diversity index, Table 3) and lowest in non-reproductive females from Herslev.

**Table 3:** Epigenetic diversity in the samples (using Mixed scoring 2 [28]).

Type of marker*	Sample	Total number of markers	% (and number) of polymorphic markers	% of private markers	Shannon’s diversity
u	Herslev_B	159	80.5 (128)	0.63	0.510
	Herlev_nr	159	71.7 (117)	0.00	0.496
	Rømø _P	159	87.4 (141)	3.77	0.650
	Rømø _nr	159	88.0 (140)	0.63	0.670
m	Herslev_B	150	72.7 (109)	4.00	0.460
	Herlev_nr	150	55.3 (83)	0.67	0.383
	Rømø _P	150	70.7 (106)	8.00	0.480
	Rømø _nr	150	77.3 (116)	9.33	0.530

*u = unmethylated, m = full or hemimethylation of the internal cytosine (B = benthic, P = planktonic, nr = non-reproductive)

### CpG o/e ratios

**Fig 1:**
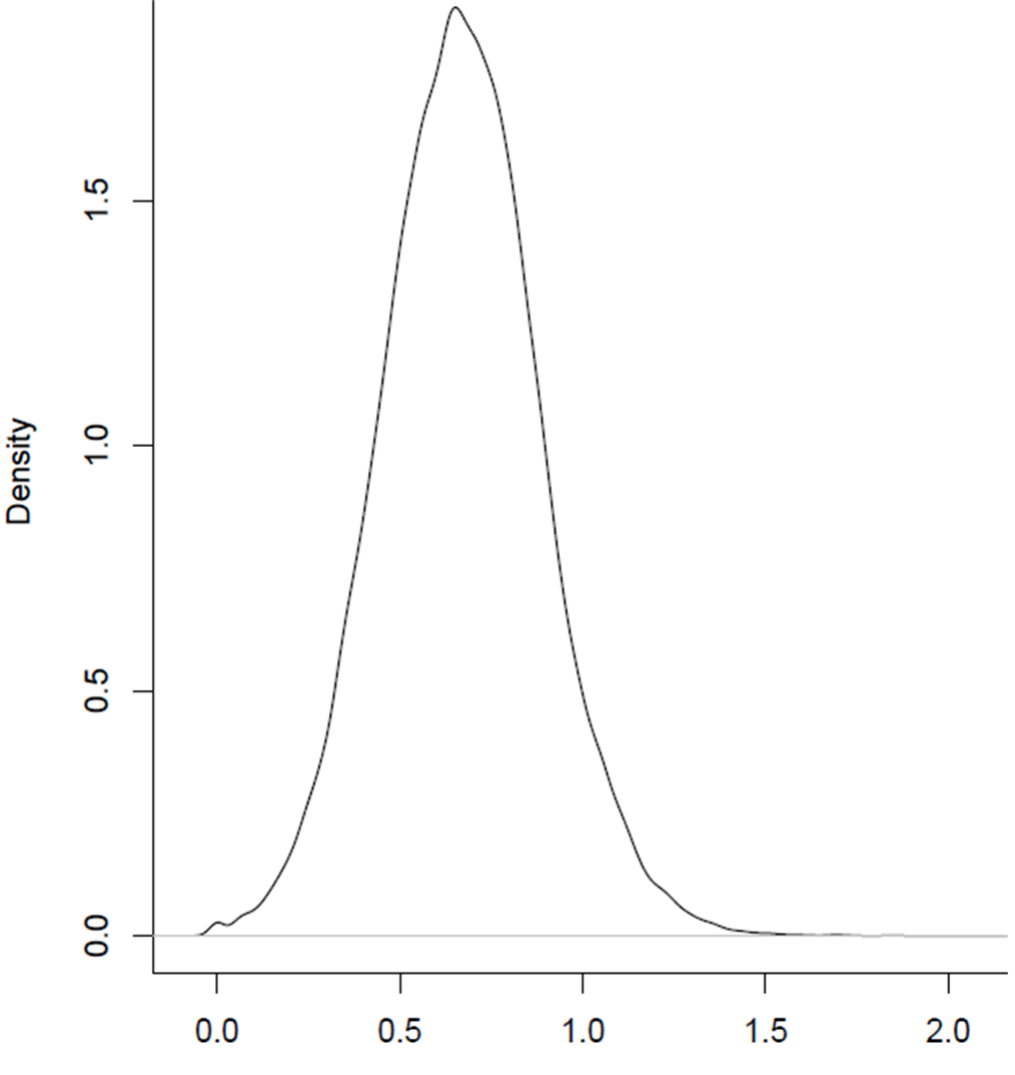
Distribution of CpG o/e ratio from all ORF containing contigs (80853) from *P*. *elegans* (mean 0.669).

The observed/expected ratio of CpG sites was calculated from the *P*. *elegans* draft transcriptome to look for signatures of intragenic DNA methylation in this species. Low CpG o/e ratio is interpreted as a sign of high persistence of (germline) methylation, whereas a high CpG o/e ratio is expected for genome regions or genes with low historical methylation (e.g. [43, 44]). Species completely lacking or having low methylation in the germline typically show a high CpG o/e ratio (>1). In *P*. *elegans*, the distribution of CpG o/e in predicted ORF containing contigs shows a unimodal distribution with an overall mean of 0.669 (Fig 1). For comparison, the probability density function for GpC o/e ratio was calculated. A unimodal normal distribution with mean approaching 1 was observed (mean = 0.960), indicating that the CpG depletion observed in *P*. *elegans* is likely due to presence of DNA methylation and that the distribution of GpC o/e is not affected by possible biases in the G+C content of the contigs. In addition, the ratio of observed and expected TpG dinucleotides shows a significant negative correlation with CpG o/e (r = -0.44, P < 2.2e-16), indicating that depletion of CpG dinucleotides is associated with an increase in TpG dinucleotides (mean for distribution of TpG o/e ratio was 1.318). Since the mean CpG o/e value seen in *P*. *elegans* transcripts is less than 1, we can conclude that methylation is present and has left an evolutionary footprint in the genome.

In order to examine differential methylation between transcripts of *P*. *elegans* involved in discrete biological processes, each of the 8658 ORF containing contigs were assigned to a GO Slim category. In the whole set of transcripts, significant differences between CpG o/e ratios of genes in different biological processes were for those involved in ‘cell adhesion’ compared to ‘cell cycle’ (P = 0.029) and for those involved in ‘death’ compared to those involved in ‘protein metabolism’ (P = 0.003), ‘RNA metabolism’ (P = 0.021), 'cell adhesion' (P = 0.001), and 'stress response' (P = 0.033) (Fig B and Table A in S1 File). In all cases genes involved in ‘death’ had lower CpG o/e ratios, i.e. higher historical methylation, than the genes in the other processes.

**Fig 2:**
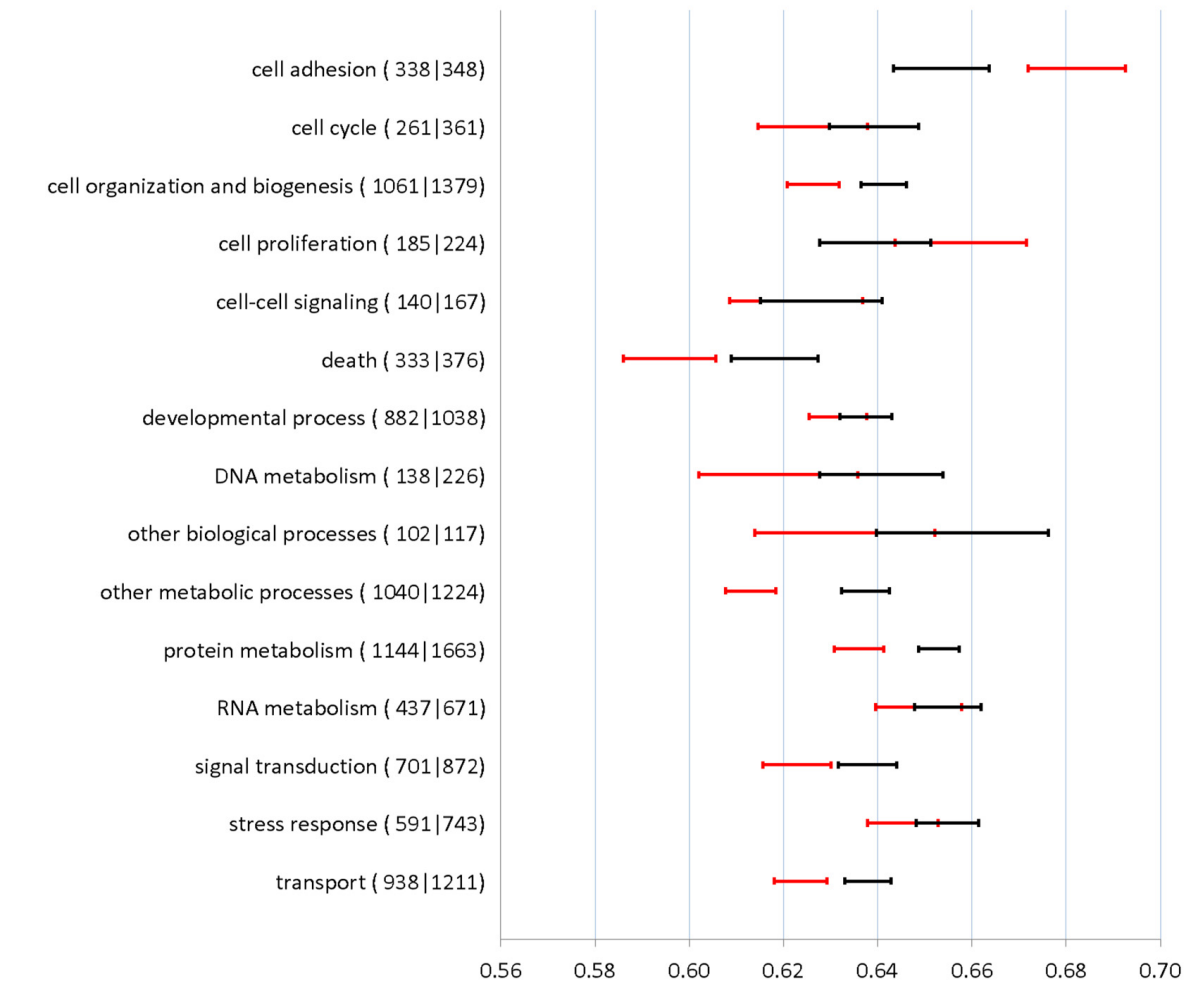
Differential methylation (measured by CpG o/e ratio) in gravid females that produce planktonic or benthic larvae. The numbers of ORF containing contigs in each GO Slim category are shown in parenthesis after category name (benthic | planktonic: total 8658). Bars represent mean CpG o/e ratio for ORF containing contigs in each GO Slim category ± 1 standard error of the mean (planktonic, black; benthic, red).

Fig 2 shows the comparison of CpG o/e ratios of transcripts in different GO Slim categories between gravid females that produce benthic larvae and gravid females that produce planktonic larvae. Significant differences were seen only in ‘protein metabolism’ (P = 0.012), ‘cell adhesion’ (P = 0.047), ‘cell organization & biogenesis’ (P = 0.042) and ‘other metabolic processes’ (P = 0.001) (Table B in S1 File). Here, females with benthic larvae were expressing transcripts with higher CpG o/e ratio in the category of ‘cell adhesion’, whereas in the other three categories, females with planktonic larvae were expressing transcripts with higher ratios. Also, the overall pattern of CpG o/e ratio of the expressed genes is higher in females with planktonic larvae (13 of 15 GO Slim categories), with exceptions in ‘cell adhesion’ and ‘cell proliferation’ (the latter had no significant difference in CpG o/e ratios between the groups).

### Genes involved in DNA methylation machinery

We searched for genes suggested to be involved in the methylation machinery from the transcriptome of *P*. *elegans* (S2 File). Transcripts of genes from each of the searched protein families (DNA methyltransferases [Dnmt], Methyl-CpG Binding Domain proteins [MBD] and Ten Eleven Translocation enzymes [TET]) were identified in a draft transcriptome of *P*. *elegans*.

For *Dnmt1*, two *P*. *elegans* contigs captured most of the conserved domains found in the N-terminal regulatory region of *H*. *sapiens Dnmt1* [37]. PYGEL76071 sequence contained three conserved *Dnmt1* domains (DNMT1-RFD, CXXC-zf and BAH_Dnmt1_I). In addition, PYGEL76073 contained three different methyltransferase specific domains (BAH_Dnmt1_I, BAH_Dnmt1_II and dcm: DNA-cytosine methylase) located at the end of the N-terminal region. These two *P*. *elegans* sequences do not overlap, but are likely two partial transcripts from the same gene (since they match to different parts of the *H*. *sapiens* query sequence, see S2 File). Both of these sequences match well to *Dnmt1* orthologs from other species (using blastp) and phylogenetic analyses support a conclusion that the contig is *Dnmt1*. In the NJ tree, sequences from vertebrates form a separate, well supported group that does not include any sequences from invertebrates. The sequence found in *P*. *elegans* (PYGEL76071, containing the conservative DNMT1-RFD domain) shows similarity to *Dnmt1* from other marine invertebrates (Fig C in S1 File). When using *Dnmt2* sequence as query, a match was found to a sequence (PYGEL58442) which contains a domain from the AdoMet_MTase super family (more specifically, a DNA methylase domain found in the C-terminal part of methyltransferases). With blastp searches, this sequence showed a good match to invertebrate tRNA cytosine-5-methyltransferase (*Dnmt2*). When using *Dnmt3* sequences as query, we found sequences in the transcriptome of *P*. *elegans* containing both of the conserved domains found in the *Dnmt3a* and *Dnmt3b* regulatory region (PWWP and ATRX-Homology domain, [37]). PYGEL17511 contained a Dnmt3b_related PWWP domain, and PYGEL118584 had an ADDz zinc finger domain (ATRX domain present in cytosine-5-methyltransferase 3). However, no clear matches to *Dnmt3* homologs from other species were found when using these *P*. *elegans* contigs as query in blastp searches, suggesting that additional data would be needed to confirm the presence of Dnmt3 proteins in *P*. *elegans*.

In mammals, multiple genes with functional methyl-CpG-binding domains (MBD) are found [6, 39], whereas in invertebrates, there is commonly only one gene (*MBD2/3*, combining the functions of *MBD2* and *MBD3*, e.g. [38]). Using *H*. *sapiens* MBD protein sequences (*MBD2*,*3*,*4*) as query, MBD domain containing sequences were also found in the *P*. *elegans* transcriptome. Sequence PYGEL94139 contained two domains from a MBD enzyme (MBD methyl-CpG-binding domain and a C-terminal domain of methyl-CpG binding protein 2 and 3). Using this sequence as query in a blastp search, the best hits are to invertebrate methyl-CpG-binding domain protein 2 or 2/3. Phylogenetic reconstructions using NJ supported orthology of this *P*. *elegans* MBD sequence to methyl-CpG-binding domain proteins. Vertebrate *MBD2* and *MBD3* proteins form their own well supported clade separated from invertebrate *MBD2/3* sequences. PYGEL94139 sequence was most similar to a predicted MBD like protein sequence from another annelid *Capitella teleta*. Furthermore, sequences from the Lophotrochozoa (mollusks, annelids, brachiopod) grouped separately from those from Arthropoda used in the phylogenetic analyses (Fig D in S1 File). Another contig (PYGEL76757) containing a conserved methyl-CpG-binding domain showed blastp hits to *MBD2*. Several matches to TETs (methylcytosine dioxygenases) were found using *H*. *sapiens* TETs as query. Several contigs, e.g. PYGEL82188 and PYGEL4276 contained the conservative oxygenase domain of the 2OGFeDO superfamily (Tet_JBP), and blastp search showed that they matched well to *TET* methylcytosine dioxygenase proteins from other species. Phylogenetic NJ analysis groups the *P*. *elegans* sequence (PYGEL82188) with predicted TET protein sequences of mollusks, whereas insect samples group together, and mammal TET sequences form their own well supported clade, as expected (Fig E in S1 File).

## Discussion

Epigenetic variation, especially DNA methylation and its functions, has been widely studied in vertebrate model species [3, 6, 37, 45]. Moreover, there is high variation in the levels and (genomic) position of CpG site methylation in different groups of organisms (e.g. [46, 47]), and some species even lack CpG site methylation [48]. In mammals and many other vertebrates, CpG methylation exists at high levels and is globally distributed in the genome, whereas in several invertebrate species, a mosaic pattern is seen with some regions of genome densely methylated and other regions lacking methylation [3, 6, 7, 35, 46, 49]. Also, overall levels of DNA methylation are generally lower in invertebrates than in vertebrates [2, 3, 33, 44, 50]. However, patterns of DNA methylation have not been investigated in most invertebrate species, and for example, only a few (annelids or) polychaetes have been studied to date (*Chaetopterus variopedatus* embryos [51] and *Spiophanes tcherniai* [10] have CpG site methylation). Additionally, the possibility for epigenetic regulation through histone modifications has been studied in the spionid *Polydora cornuta* [52]. Information about the presence DNA methylation in a broader range of invertebrate species will help advance understanding the importance of epigenetic variation in invertebrate genomes.

We examined the presence of DNA methylation in the spionid polychaete *Pygospio elegans*, a poecilogonous species with females that can produce different types of larvae [18–20]. How poecilogony is possible in this species is not clear, but the different larval phenotypes could result from possible fixed genetic differences, environmentally induced plasticity, epigenetic or maternal effects (reviewed in [53]). Investigating the presence of DNA methylation in *P*. *elegans* is an important first step in determining whether epigenetic regulation via changes in DNA methylation, possibly leading to changes in gene expression, could have a role in the observed differences in maternal provisioning and larval phenotype evident in the different larval developmental modes.

Bisulfite treatment and sequencing of a fragment of Histone H3 gene revealed the presence of intragenic CpG site methylation in *P*. *elegans*, a pattern commonly seen in other invertebrates [5, 7, 33, 34, 35,46]. Although DNA methylation in promoter regions (in vertebrates) is more often linked to silencing of gene expression, DNA methylation in coding regions also regulates gene expression levels, in particular, higher intragenic methylation levels are often correlated with higher gene expression in invertebrates [2, 3, 7, 8, 33]. In addition, patterns of methylation in coding regions is suggested to be involved in alternative splicing ([7, 8, 35], but not always, for example in *Nasonia*; [33]).

Methylation-sensitive AFLP (MS-AFLP) analysis showed differences in genome-wide CpG site DNA methylation patterns and therefore suggests epigenetic differences among our samples. Most interestingly, MS-AFLP analysis showed significantly different methylation profiles for the gravid females with different developmental modes, i.e. different loci, or genes, were methylated. In Herslev, methylation profiles between gravid females with benthic larvae and non-reproductive females were also significantly different and methylation level was higher in the gravid females (using both scoring approaches for the MS-AFLP data). However, no differences were seen in Rømø, despite reproductive status of the individuals. In addition, the non-reproductive females from the two sites (Herslev & Rømø) also showed differentiation in their methylation profiles, which could be associated with possible genetic differences between the sampled populations.

Even though our results with the methylation sensitive restriction enzymes are indicative of genome-wide patterns, the AFLP-based analysis only captures part of the variation of the genome, and only methylation patterns at the 5’- CCGG -3’ restriction site are seen. In other studies using the MS-AFLP method, clear epigenetic differences have been found in tissue specific analyses (e.g. gonad and brain) between mature and immature Atlantic salmon [54] and between reproductive and non-reproductive bumble bees [55]. It is not known if *P*. *elegans* exhibits methylation in regions of the genome other than at CpG sites (as seen in some other invertebrates: [56]).

Additionally, we used transcriptome data to search for genes suggested to be involved in processes of DNA methylation. DNA methyltransferases are enzymes that establish and maintain DNA methylation of cytosines (by attaching the methyl group). In human and many other vertebrates, three subfamilies are found: *Dnmt1* is the maintenance enzyme copying methylation status of the genome during DNA replication (patterns inherited through mitosis), whereas two of the three enzymes in the Dnmt3 family (*Dnmt3a* and *Dnmt3b*) are involved in the de novo methylation of previously unmethylated sites. A third Dnmt3 protein (*Dnmt3L*) and *Dnmt2* do not have methyltransferase activity, but *Dnmt3L* interacts with the other Dnmt3 enzymes and Dnmt2 is involved in tRNA methylation (see [6, 37]). Invertebrates show variable numbers of Dnmt enzymes, and the maintenance enzyme *Dnmt1* is the most commonly found member of the gene family. In insects (reviewed in [47]), some species have lost Dnmt genes (e.g. *Drosophila melanogaster* only has *Dnmt2*, and the beetle *Tribolium castaneum* has lost all *Dnmt3* genes), whereas others have multiple copies of the *Dnmt1* gene (e.g. the wasp *Nasonia vitripennis*). In molluscs, for example, a *Dnmt3* homologue is found in *Crassostrea gigas* [36], but not in the snail *Biomphalaria glabrata* [44]. DNA methylation in polychaetes has not been widely studied, but CpG site DNA methylation (in embryos) and the likely presence of at least one DNA methyltransferase enzyme was observed in *C*. *variopedatus* [51, 57] and homologues of all three Dnmt gene families have been found in the *C*. *teleta* genome (see [47]). We found partial transcripts for *Dnmt1* (and *Dnmt2*) in the transcriptome of *P*. *elegans*. The presence of genes from the *Dnmt3* family could not be verified in this study. *Dnmt3* family enzymes are particularly relevant since it is suggested that Dnmt3s are involved in establishing new methylation patterns in response to environmental triggers [4]. More transcriptome data or preferably genome sequence information is needed to confirm our preliminary identification of *Dnmt3* genes in *P*. *elegans*.

Methyl-CpG binding proteins (MBDs) recognize and bind to methylated CpG sites and affect gene expression by blocking the binding of transcription factors or forming complexes with other proteins, such as histone modifying enzymes, to change chromatin structure and the shape of active sites [4, 6, 9]. MBDs can also interact with *Dnmt1* to affect gene expression [4]. Multiple functional MBDs are commonly found in vertebrates [6, 38, 39], whereas in invertebrates, only one MBD enzyme (*MBD2/3*) is often found [38, 44], and it is likely to be present also in *P*. *elegans*. Also, indication of the presence of a TET enzyme (methylcytosine oxygenase) is found from the *P*. *elegans* transcriptome. *TET*s are suggested to be involved in demethylation (removal of methyl groups) and regulation of DNA methylation, at least in mammals [40, 41]. One *TET* orthologue is found in many invertebrate species, and a recent study has shown that the single *TET* orthologue found in honeybee *A*. *mellifera* has oxidation activity and is capable of converting methylated cytosine to 5hmC [58]. However, the role of this protein in non-mammalian species is still largely unstudied and unclear. To summarize, partial transcripts of genes from the gene families suggested to be a part of CpG site methylation processes were found from *P*. *elegans* transcriptome data, suggesting that some crucial elements of DNA methylation machinery are present and that this species is capable of DNA methylation. Given these findings, it is possible that DNA methylation could be one mechanism for epigenetic regulation of gene expression in this species.

An analysis of observed/expected ratios of CpG sites in the *P*. *elegans* transcriptome also provided an estimation of DNA methylation patterns in this species. Low CpG o/e ratio indicates higher levels of consistent historical methylation of germline leading to depletion of CpG over time due to the higher mutation vulnerability (deamination to thymines) of methylated cytosines [31, 32, 43, 44]. High CpG o/e ratio indicates areas or genes with lower historical methylation. For example, *D*. *melanogaster* and *Anopheles gambiae* genomes show high CpG o/e ratios close to 1, indicating no CpG depletion [43]. In *P*. *elegans*, the distribution of CpG o/e in the predicted ORFs was unimodal (although the shape of the frequency curve is strongly affected by the type of data used) with a mean value much lower than 1, a signature of CpG site methylation leading to depletion of CpG sites in the genome of this species.

In the honeybee, *Apis mellifera*, a bimodal distribution of CpG o/e ratios has been observed [43, 59], with a group of unmethylated genes (CpG o/e >1) and a group of methylated genes with means of 1.50 and 0.55 using genome sequence data (bimodal distributions seen also in *C*. *gigas*: 0.70 & 0.40 [36] and *B*. *glabrata*: 0.62 & 0.21 [44], both with transcriptome data). The *A*. *mellifera* studies and other similar analyses of CpG o/e ratios in addition to bisulfite sequencing of other invertebrate species have shown that housekeeping genes and conserved proteins with constant expression are commonly found in the group of genes with low CpG o/e ratio, indicating depletion of CpG sites (due to consistent historical methylation) [7, 33, 34, 36, 43, 44, 59]. In contrast, for example in the honey bee, genes with high CpG o/e ratio (low expected germline methylation) show caste specific expression differences [43]. Additionally, other studies have shown that non-methylated genes (or genes with low germline methylation) have higher variation in gene expression (among phenotypes, samples or tissues) compared to the expression of conserved proteins with high methylation levels [7, 33, 43, 59]. Such genes with low (germline) methylation in coding regions might be involved in epigenetic regulation of phenotypic plasticity or adaptive responses of organisms through transient regulation of gene expression (if methylation is present transiently only at certain life stages or tissues). For example, genes involved in responses to stress or environmental change with no signs of CpG site depletion (i.e. no consistent historical germline methylation) suggest that transient DNA methylation might be important for regulating their transcription in response to environmental factors or triggers [9, 36, 56]. We did not find groups of high and low historically methylated genes in *P*. *elegans*: ORF containing contigs divided into groups based on discrete biological processes did not significantly differ in CpG o/e ratios, possibly due to the lack of bimodality in the distribution curve.

We did not find many significant differences in CpG o/e ratios in the transcriptomes of gravid females producing different types of larvae, however, in most gene categories, females producing planktonic larvae were expressing transcripts with higher CpG o/e ratio (indicating gene transcripts with lower germline methylation). This is interesting, since the MS-AFLP analysis indicated similar overall levels of methylation in the genomic DNA of both types of gravid females (those producing planktonic larvae and those producing benthic larvae). However, differences in the methylation profiles between the types were also observed, and this could explain the different pattern seen at the gene expression level (different genes or transcripts with different CpG content were expressed). In the future, it would be interesting to compare the different gravid females by comparing differentially expressed genes only, to clarify if genes associated with developmental mode differences also differ in CpG o/e ratios and methylation levels. Epigenetic regulation of differentially expressed genes might allow for higher phenotypic plasticity if epigenetic patterns are changed (transiently) in response to external factors [60]. Additionally, performing whole genome bisulfite sequencing on *P*. *elegans* would reveal more directly the degree to which different genes are methylated.

To conclude, we found that intragenic CpG site DNA methylation and some of the genes of methylation machinery are present in the *P*. *elegans* genome, as has been observed for some other marine invertebrates. However, more studies are needed, for example some genes involved in the methylation pathway may not have been captured by our transcriptomic data set, and these could be identified if genome data becomes available. Also, other epigenetic changes not studied here, such as histone modifications or microRNAs could be used in epigenetic regulation of gene expression in this species. In addition, we found differences in CpG site methylation patterns between most of our samples: MS-AFLP method showed significant differences between methylation profiles of females producing larvae destined to different developmental modes, and gravid females from Herslev producing benthic offspring show a different methylation profile in comparison to non-reproductive females from the same population. Additionally, CpG o/e ratios of expressed genes give indications that gravid females producing the different larval types are expressing transcripts with differences in their CpG site content (i.e. differences in gene expression profiles are suggested). These indications of differences in DNA methylation patterns suggest a potential for epigenetic regulation of developmental mode.

## Supporting Information

S1 File Additional information on CpG site methylation confirmation (bisulfite treatment), phylogenetic analyses, CpG o/e ratio analyses and primers used in MS-AFLP. (PDF)

S2 File Genes involved in DNA methylation machinery. This file contains detailed results of the blast searches and conserved domains search for the genes involved in DNA methylation machinery.
(XLSX)
