# Supplementary material for: DNA Methylation and Potential for Epigenetic Regulation in Pygospio elegans
Source: PLoS One. 2016 Mar 23;11(3):e0151863. doi: 10.1371/journal.pone.0151863 (PMC4805255; doi:10.1371/journal.pone.0151863)
Supplement: S1 File — (PDF) [file pone.0151863.s001.pdf]

Supporting information S1.

Figure A. Histone H3 sequence alignment (untreated and bisulfite treated sequence) to detect methylated cytosines.

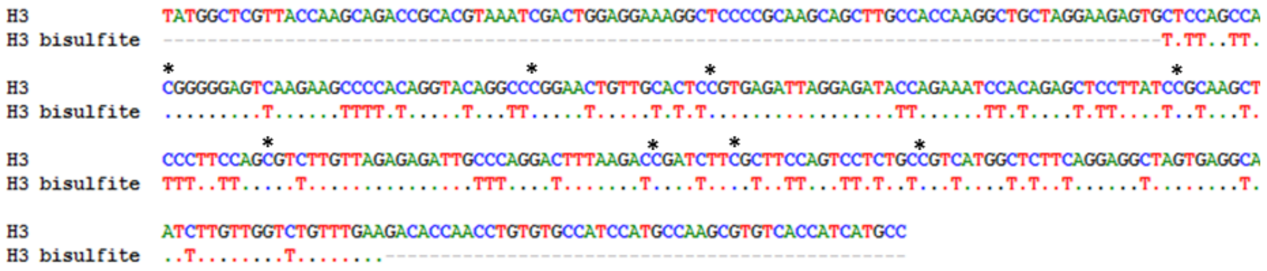

A 367 bp fragment of the histone H3 (H3) gene from *Pygospio elegans* aligned with a 230 bp sequence obtained from the same DNA treated with bisulfite conversion. In the H3 bisulfite sequence, dots (.) are used when the sequence is identical to the untreated sequence above, T is for sites where unmethylated cytosines have been converted to thymines, and asterisks (*) are used to point out the eight methylated cytosines which have not been converted during the bisulfite treatment (all at CpG sites).

Figure B. Variation in methylation (measured by CpG o/e ratio) of 8658 ORF containing transcripts from *P. elegans* assigned to discrete biological processes. The number of contigs in each category is shown in parenthesis after the category name. Bars represent mean CpG o/e ratio for ORF containing contigs in each GO Slim category ± 1 standard error of the mean.

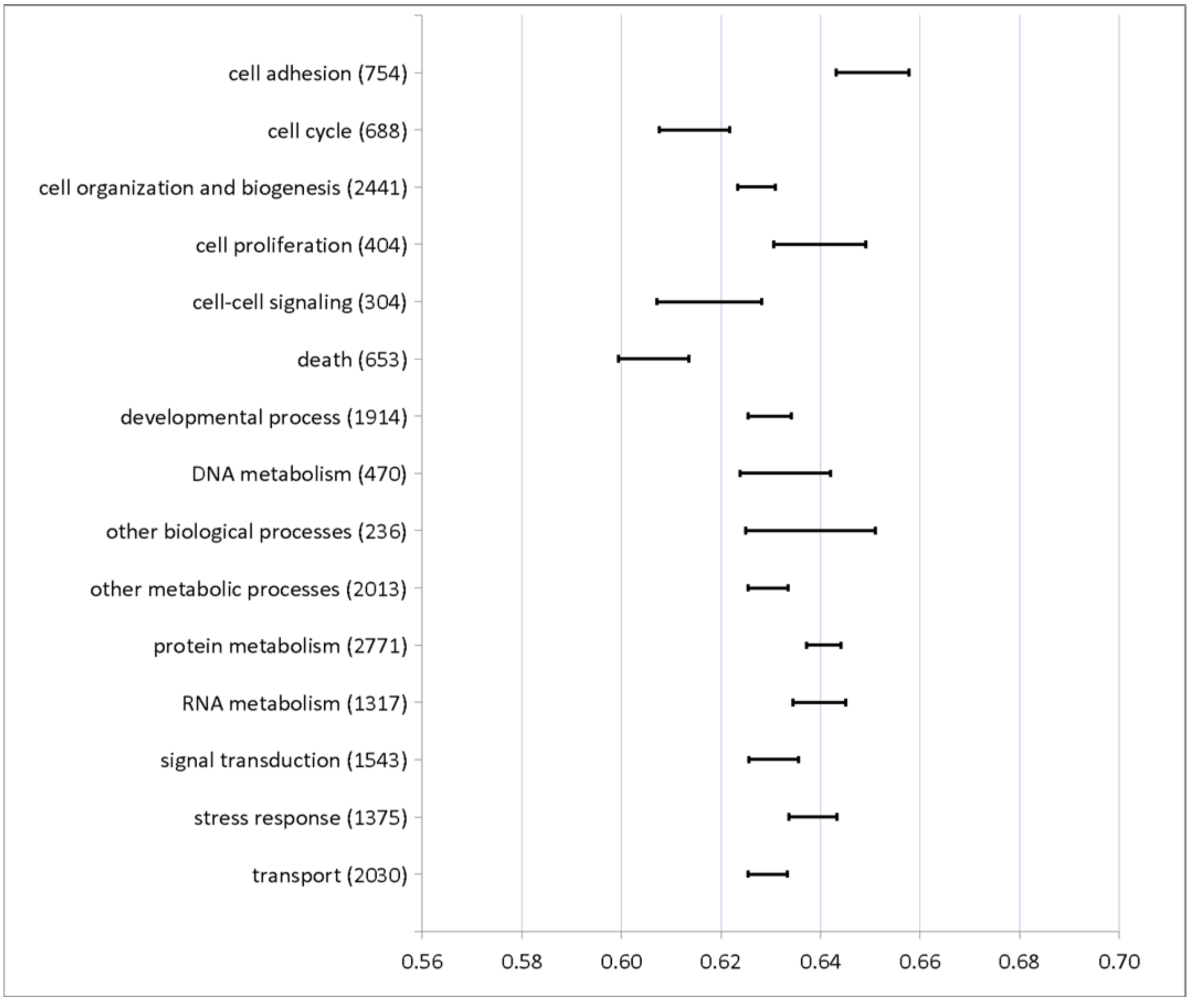

Figure C. Neighbor-joining phylogenetic tree for Dnmt1 sequences (with bootstrap support values of >70% on the nodes).

Species and accession numbers: *Bombyx mori* (NP_001036980.1), *Daphnia pulex* (EFX80183.1), *Nasonia vitripennis* (NP_001164521.1), *Apis mellifera* (XP_006562865.1), *Helobdella robusta* (ESN92163.1), *Aplysia californica* (XP_012936221.1, XP_005095277.1), *Biomphalaria glabrata* (XP_013075419.1), *Crassostrea gigas* (EKC23761.1), *Capitella teleta* (ELT93682.1), *Danio rerio* (NP_571264.2), *Homo sapiens* (AAI26228.1), *Mus musculus* (AAH53047.1)

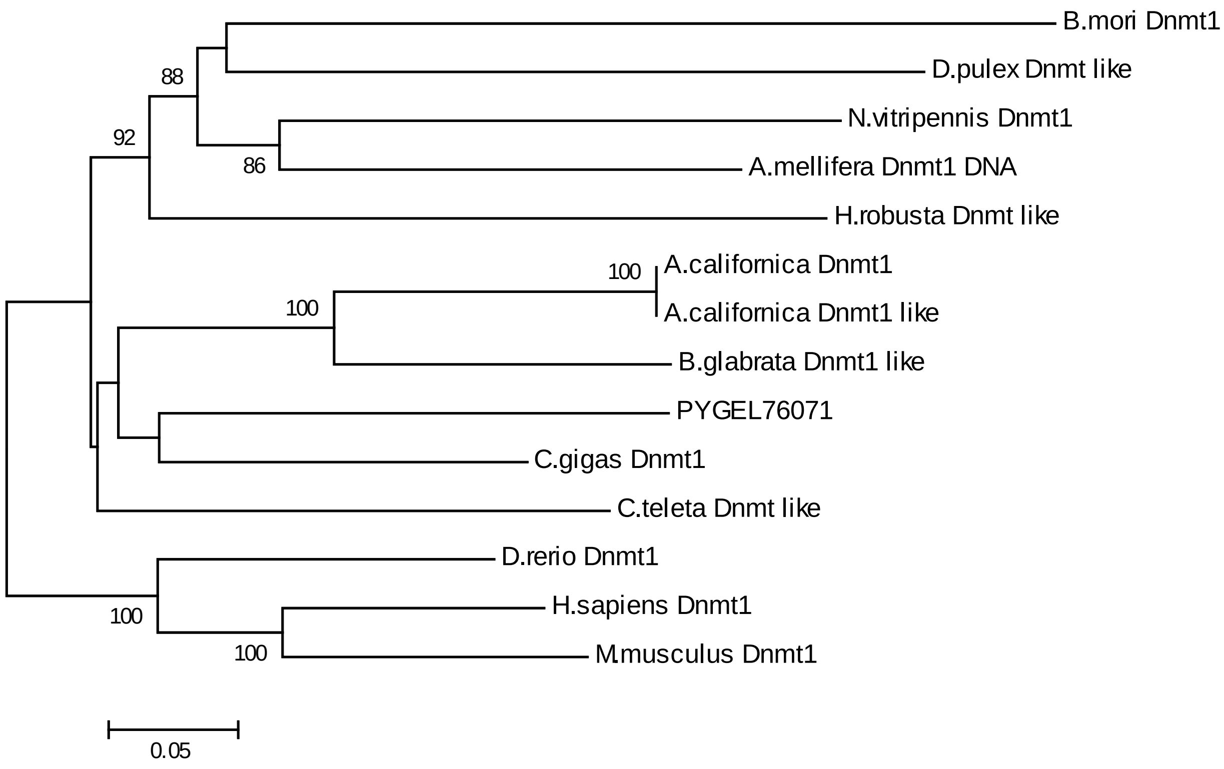

Figure D. Neighbor-joining phylogenetic tree for MBD sequences (MBD2, 3 & 2/3) (with bootstrap support values of >70% on the nodes).

Species and accession numbers: *Aplysia californica* (XP_005103642.1), *Biomphalaria glabrata* (NP_001298225.1), *Crassostrea gigas* (EKC32831.1), *Lottia gigantea* (XP_009048664.1), *Lingula anatina* (XP_013380854.1), *Capitellla teleta* (ELU03991.1), *Ixodes scapularis* (XP_002407962.1), *Daphnia pulex* (EFX83334.1), *Apis mellifera* (XP_392422.2), *Drosophila melanogaster* (AF171098_1), *Homo sapiens* (MBD1: NP_001191066.1, MBD2: AAC68871.1, MBD3: AAC68876.1), *Danio rerio* (MBD2: NP_997933.1, MBD3: NP_997934.1), *Mus musculus* (MBD2: NP_034903.2, MBD3: NP_038623.1), *Xenopus laevis* (NP_001083787.1)

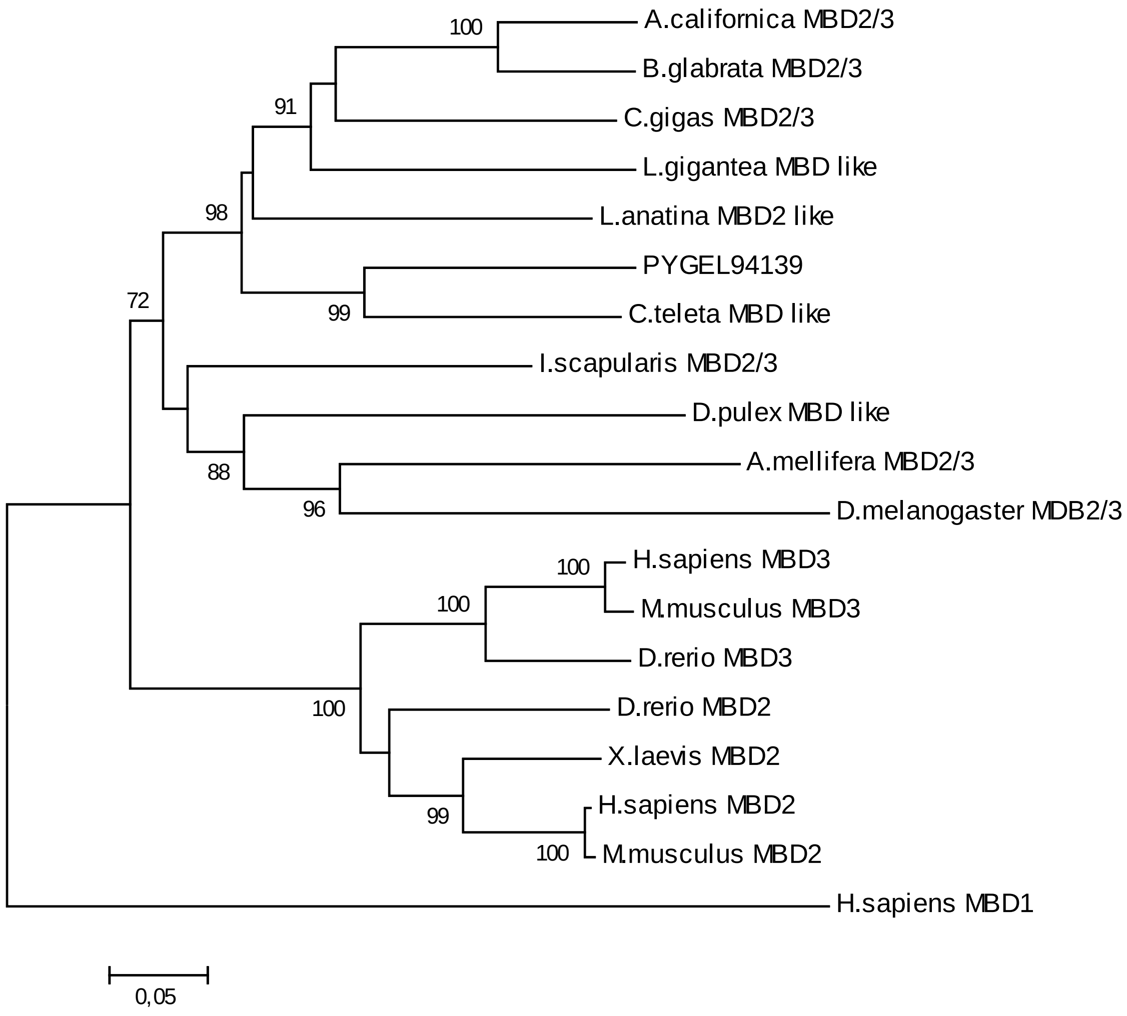

Figure E. Neighbor-joining phylogenetic tree for TET sequences (with bootstrap support values of >70% on the nodes).

Species and accession numbers: *Crassostrea gigas* (XP_011419066.1, EKC18772.1), *Lottia gigantea* (XP_009060167.1), *Biomphalaria glabrata* (XP_013078194.1), *Aplysia californica* (XP_005092662.1), *Capitella teleta* (ELU00383.1), *Drosophila melanogaster* (NP_001246581.1), *Nasonia vitripennis* (XP_008203342.1), *Apis mellifera* (XP_006561262.1), *Melipona quadrifasciata* (KOX75173.1), *Habropoda laboriosa* (KOC60806.1), *Homo sapiens* (TET1: NP_085128.2, TET2: NP_001120680.1, TET3: NP_001274420.1), *Mus musculus* (NP_898961.2)

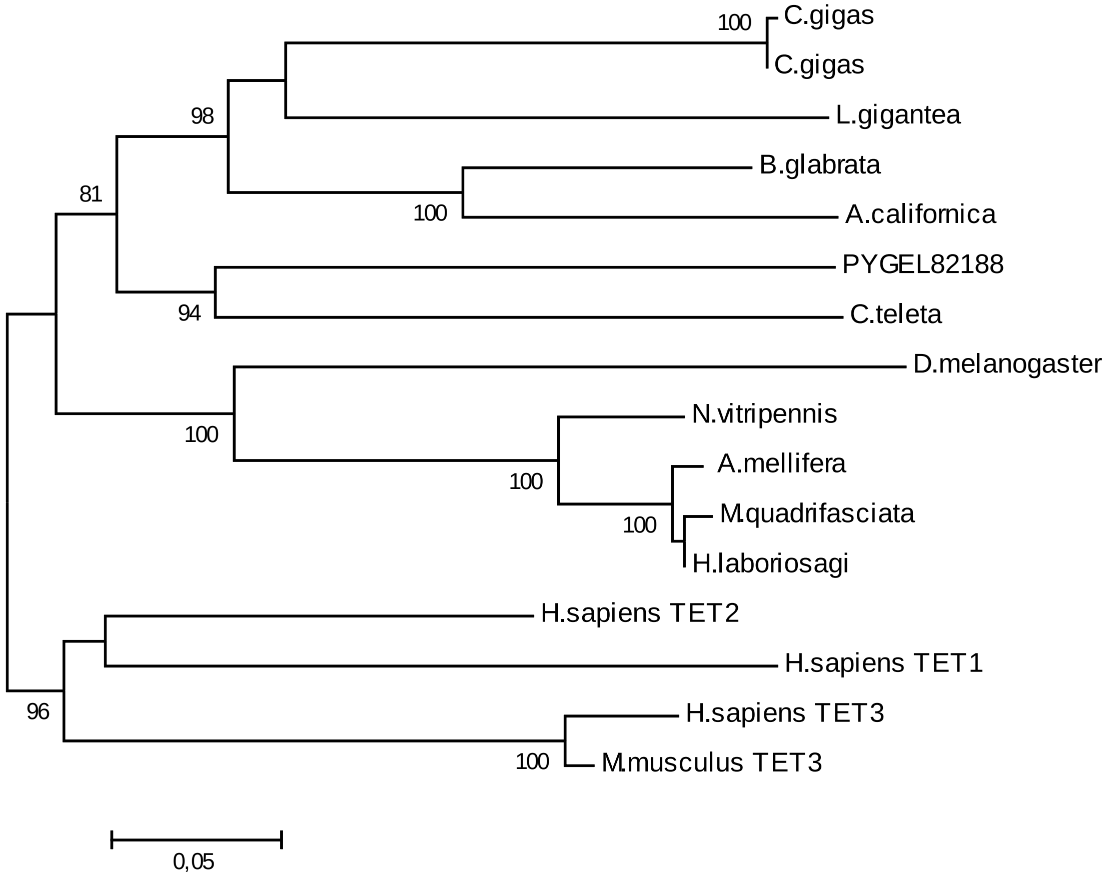

Table A. p-values for difference of methylation between *P. elegans* transcripts involved in discrete biological processes (measured by CpG o/e). All 8658 ORF containing contigs included. Significant values between category mean CpG o/e (< 0.05) are underlined.

	cell adhesion	cell-cell signaling	cell cycle	cell organization and biogenesis	cell proliferation	death	developmental process	DNA metabolism	other biological processes	other metabolic processes	protein metabolism	RNA metabolism	signal transduction	stress response
cell-cell signaling	1.00	-	-	-	-	-	-	-	-	-	-	-	-	-
cell cycle	<u>0.029</u>	1.00	-	-	-	-	-	-	-	-	-	-	-	-
cell organization and biogenesis	0.291	1.00	1.00	-	-	-	-	-	-	-	-	-	-	-
cell proliferation	1.00	1.00	1.00	1.00	-	-	-	-	-	-	-	-	-	-
death	<u>0.001</u>	1.00	1.00	1.00	0.501	-	-	-	-	-	-	-	-	-
developmental process	1.00	1.00	1.00	1.00	1.00	0.608	-	-	-	-	-	-	-	-
DNA metabolism	1.00	1.00	1.00	1.00	1.00	1.00	1.00	-	-	-	-	-	-	-
other biological processes	1.00	1.00	1.00	1.00	1.00	1.00	1.00	1.00	-	-	-	-	-	-
other metabolic processes	0.907	1.00	1.00	1.00	1.00	0.6418	1.00	1.00	1.00	-	-	-	-	-
protein metabolism	1.00	1.00	0.111	0.965	1.00	<u>0.003</u>	1.00	1.00	1.00	1.00	-	-	-	-
RNA metabolism	1.00	1.00	0.443	1.00	1.00	<u>0.021</u>	1.00	1.00	1.00	1.00	1.00	-	-	-
signal transduction	1.00	1.00	1.00	1.00	1.00	0.5976	1.00	1.00	1.00	1.00	1.00	1.00	-	-
stress response	1.00	1.00	0.655	1.00	1.00	<u>0.033</u>	1.00	1.00	1.00	1.00	1.00	1.00	1.00	-
transport	0.859	1.00	1.00	1.00	1.00	0.6652	1.00	1.00	1.00	1.00	1.00	1.00	1.00	1.00

Table B. p-values (significance threshold < 0.05) for comparisons between GO Slim categories for benthic vs. planktonic gravid females. Comparison is based on difference in CpG o/e ratios.

**GO category (#ORF benthic|planktonic)**	p-value
cell adhesion (338|348)	0.04716
cell cycle (261|361)	0.38904
cell organization and biogenesis (1061|1379)	0.04173
cell proliferation (185|224)	0.31999
cell-cell signaling (140|167)	0.78075
death (333|376)	0.09763
developmental process (882|1038)	0.46346
DNA metabolism (138|226)	0.30446
other biological processes (102|117)	0.34460
other metabolic processes (1040|1224)	0.00104
protein metabolism (1144|1663)	0.01190
RNA metabolism (437|671)	0.58868
signal transduction (701|872)	0.11549
stress response (591|743)	0.34359
transport (938|1211)	0.05586

Table C. Primers used in the selective PCR of the methylation-sensitive AFLP protocol

Six different primer combinations used in the selective PCR of the MS-AFLP protocol, number of fragments (loci) produced by each primer pair, number of methylation-sensitive loci (MSL), estimated with the msap program.

EcoRI	HpaII/MspI	Fragments	MSL
5'–GACTGCGTACCAATTC**ACA**–3'	5'–GATGAGTCTAGAACGG**TGTT**–3'	22	13
5'–GACTGCGTACCAATTC**ACA**–3	5'–GATGAGTCTAGAACGG**TAC**–3'	33	14
5'–GACTGCGTACCAATTC**AGA**–3'	5'–GATGAGTCTAGAACGG**TAC**–3'	26	15
5'–GACTGCGTACCAATTC**ATC**–3'	5'–GATGAGTCTAGAACGG**TTA**–3'	31	16
5'–GACTGCGTACCAATTC**ATC**–3'	5'–GATGAGTCTAGAACGG**TGTT**–3'	25	19
5'–GACTGCGTACCAATTC**ATC**–3'	5'–GATGAGTCTAGAACGG**TAC**–3'	28	21
